# Non-invasive physical plasma improves conventional wound management of cut and bite wounds in wild European hedgehogs

**DOI:** 10.1038/s41598-025-86376-2

**Published:** 2025-01-22

**Authors:** Jürgen Eichler, Björn Rulik, Alexander Abazid, Matthias B. Stope

**Affiliations:** 1 Tierarztpraxis im Frauenviertel, Small Animal Veterinary Practice, Berlin, Germany; 2 Zoological Research Museum Alexander Koenig, Leibniz Institute for the Analysis of Biodiversity Change, Bonn, Germany; 3 Department of General, Visceral and Thorax Surgery, Bundeswehr Hospital Berlin, Berlin, Germany; 4 Physical Plasma Medicine Laboratories, Department of Gynecology and Gynecological Oncology, University Hospital Bonn, Bonn, Germany

**Keywords:** Cold plasma, Cold atmospheric plasma, Tissue tolerable plasma, Plasma medicine, Physical plasma medicine, Wound healing, Veterinary medicine, Veterinary research, Wildlife biology, Zoology, Diseases

## Abstract

Non-invasive physical plasma (NIPP) has been used effectively for wound healing in human medicine for over two decades. The advantages are that NIPP has few side effects, is painless and gentle on the tissue. The therapeutic effect is mediated by reactive oxygen species (ROS). Based on the biomedical effects known to date, it can be assumed that NIPP can also be used for wound treatment in non-human mammals. In this prospective, non-randomized monocentric clinical trial, 43 European hedgehogs with cut and bite wounds were treated with conventional wound management (CWM: 21 patients) and compared with 22 patients with CWM plus NIPP treatment (CWM + NIPP). Under NIPP treatment, patients showed no signs of pain, stress or discomfort, even after several applications. In 76% of CWM + NIPP patients, three or four NIPP applications were sufficient. In patients in the CWM + NIPP group, wound treatment was completed statistically significantly 6 d earlier (CWM: 19.0 d versus CWM + NIPP: 13.2 d; *p* = 0.0008). This wildlife clinical trial demonstrates that NIPP can be used to improve wound healing in wild European hedgehogs. It is conceivable that NIPP therapy could also lead to positive effects in other injured wild animals, domestic animals or livestock.

## Introduction

The Western European hedgehog is found in the primarily urban habitats of Central, Southern and Northern Europe^1^. European Hedgehog populations are declining across Europe^2^. This is partly due to biological and anthropogenic factors that can lead to injury and disease^3–7^. In addition to animal bites, road kills and lawn mowing in urban habitats often lead to the injury and death of European hedgehogs^8–10^.

Sick and injured animals are frequently cared for in wildlife rescue centers under veterinary supervision. European Hedgehogs are one of the most common species to be handed in^11,12^. This is due to its wide distribution in densely populated areas. In addition, sick and injured European hedgehogs are often easier to find than other wild animal species that retreat and hide. Such animals are usually in a poor general condition and need to be cared for. Practically every European hedgehog is infected or infested with parasites. In the case of wounded or weakened animals, fly larvae are also often found in the wounds or body orifices. Therefore, the animals usually require veterinary care beyond the primary injury/illness. The most common injuries include cuts and bites, which require appropriate wound medical care^6,7,9,10^. The mortality rate of European hedgehogs is relatively high and can reach up to 80% in winter and in young animals^13^.

The biomedical efficacy of non-invasive physical plasma (NIPP) is diverse and ranges from vitalizing effects (proliferation, anti-apoptosis, motility, increased metabolism, immune modulation) to devitalizing phenomena (growth inhibition, antibiosis, apoptosis induction, necrosis, inhibition of cell migration). The effect depends on device parameters and treatment time and distance, but also significantly on the treated tissue^14–19^. In human medicine, NIPP has been used for over two decades for the treatment of acute and chronic wounds, particularly in cases of wound healing disorders. Pathophysiologically, NIPP-induced modulation of interleukins, angiogenesis factors, motility factors and the extracellular matrix plays a significant role here^20–23^. Clinical trials confirm the positive effects of NIPP in wound therapy in human patients^24–26^. Additionally, the anti-microbial effect of NIPP also comes to the foreground^27^.

We have already been able to demonstrate the positive effect of the combination of conventional wound care and NIPP therapy on a single injured European hedgehog^28^. This present clinical trial is a proof-of-principle study for evaluation. The control group underwent conventional wound care (CWM), while the NIPP intervention group also received NIPP therapy (CWM + NIPP). The aim of the study was to evaluate the wound healing-promoting effect of NIPP therapy on cut and bite wounds of injured European hedgehogs. The application of NIPP for wound healing and regeneration of tissue lesions in classical veterinary medicine, but also in zoo and wildlife medicine, appears promising. However, there have been hardly any systematic studies to date. A systematic literature search was therefore carried out.

## Materials and methods

### Study design

The present study was designed and implemented as a prospective, non-randomized and monocentric clinical trial. The study center was a non-governmental, donor-funded wildlife rescue center in North Rhine-Westphalia, Germany. Injured European hedgehogs were admitted under veterinary care and received medical therapy. The wild animals were usually found in public by walkers or garden owners and taken in for veterinary care. The injuries and the clinical picture of the individual patients therefore varied, and a medical history was not available.

Due to the unpredictable patient numbers, no case number estimation was performed. In order to obtain a homogeneous and seasonally independent patient distribution for the CWM group and the CWM + NIPP group, the cases of one year were combined in one group. From April 2021 to Aug 2021 the CWM patients were enrolled, from May 2022 to Nov 2022 the CWM + NIPP treatments were performed. There were two reasons for this temporal distinction between the control and intervention group. On the one hand, the availability of the NIPP device was limited to 1 year. On the other hand, a one-year clinical trial would have halved the number of cases. We therefore decided on a two-year trial period.

Inclusion criteria were cuts or bites to the head, body or extremities. In the case of multiple wounds, the wound location was assigned to the body area in which the largest wound area occurred. Comorbidities such as microbial infection and parasite infestation were treated in parallel and did not lead to exclusion from the study. The outcome of the clinical trial was determined by the number of days until successful completion of wound treatment. Wound treatment was completed when the wound surface was completely reepithelialized and no signs of complications were visible, e.g. fluid secretion. This endpoint also applied if comorbidities required further treatment. For example, it was possible that wound treatment was completed, but individual patients remained in the wildlife rescue center and were treated for parasites or malnutrition. Exclusion criteria were injuries other than cuts or bites, e.g. contusions and fractures, which are often caused by road kills. In addition, no patients with non-traumatological diseases or infections or parasite infestation as the sole disease were included in the study.

### Ethical approval

Ethical review and approval was not required for this study. Patients were delivered to the wildlife rescue center as emergencies and required veterinary care. Patient information and consent is not applicable in veterinary medicine. Informed consent from animal owners is also not applicable for wildlife. Data protection aspects therefore do not have to be taken into account either.

### Wound management

**Fig. 1:**
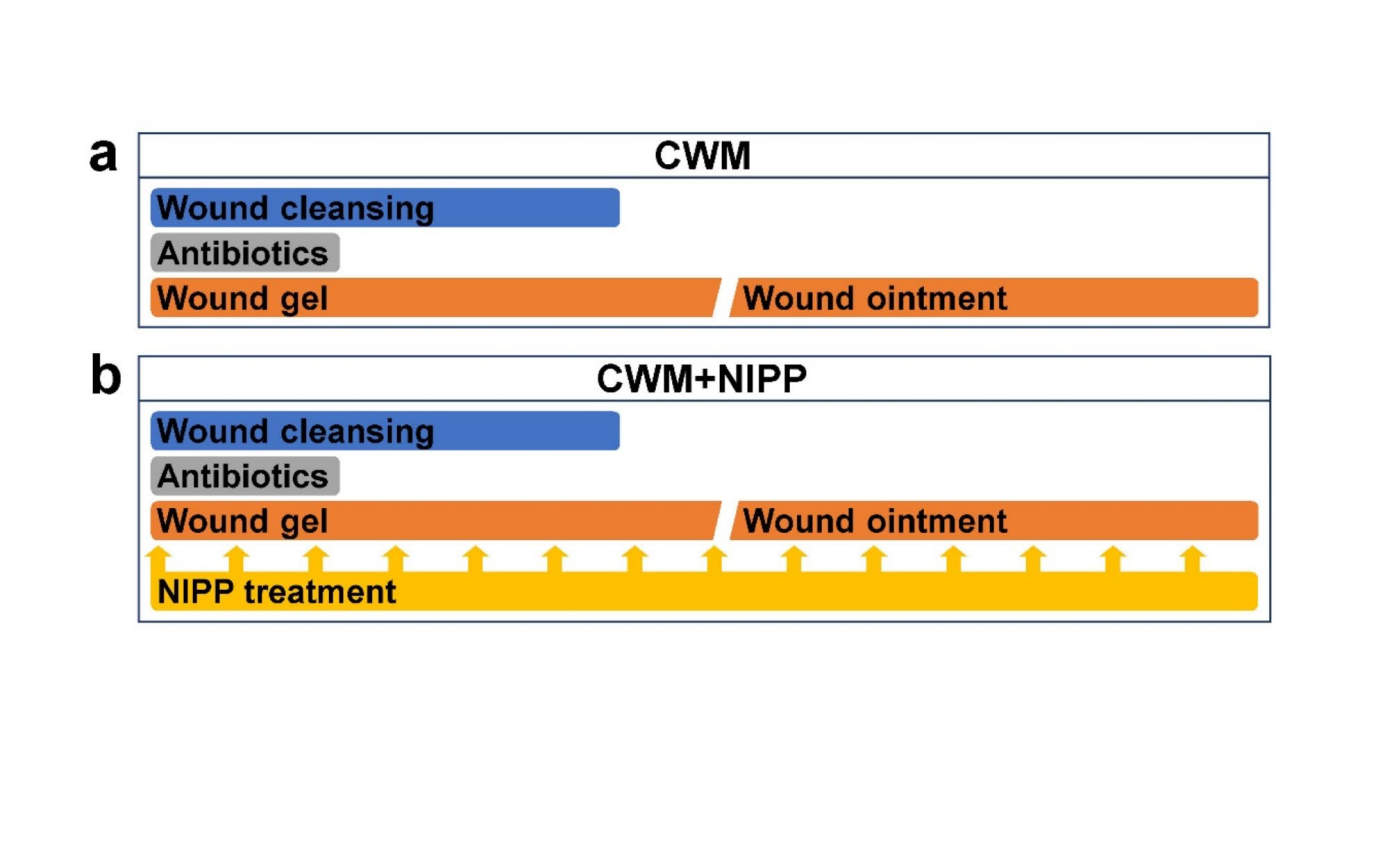
Treatment regimens of the two study cohorts. (**a**) Patients with conventional wound management (CWM) served as the control group. This consisted of wound cleansing (blue), e.g. with Ringer’s solution or hypochlorous acid solution, antibiotic administration (gray), e.g. octenidine, and wound gel (red), e.g. polihexamide. The wound gel was replaced by wound ointment (yellow), e.g. dexpanthenol, in the course of the CWM. (**b**) The intervention group CWM + NIPP received a 2 min local treatment with non-invasive physical plasma (NIPP, yellow) every 3 d in addition to CWM.

In the control group, all patients received conventional wound management (CWM, Fig. 1a). This included primary wound cleansing (Ringer’s solution or hypochlorous acid solution), disinfection (octenidine), and wound gel treatment (polihexamide gel). Wound gel was renewed daily as needed, depending on the area and the course of healing. In the last phase of reepithelialization of the wound bed, wound gel treatment was discontinued and replaced by wound ointment (dexpanthenol). In the intervention group, NIPP treatment was given every 3 days in addition to CMW (CWM + NIPP, Fig. 1b). A 2-minute NIPP treatment was carried out every 3 days. If the wound area encompassed more than the opening of the spacer of the NIPP device (16 cm^2^), treatment was performed again on the untreated wound area for 2 minutes. Patients were not anesthetized.

Intentional care was taken to ensure that patients in the CWM group and the CWM + NIPP group received identical CWM treatments as far as possible. Wound healing was considered to be completed when the wound area was fully reepithelialized and no lesions or distinctly moist areas were visible. The age of the patients was estimated by experienced veterinarians. The sex was determined on the basis of the external genitalia.

A total of 8 patients were excluded from the clinical trial and the analyses due to their poor health at the time of recruitment.

**Fig. 2:**
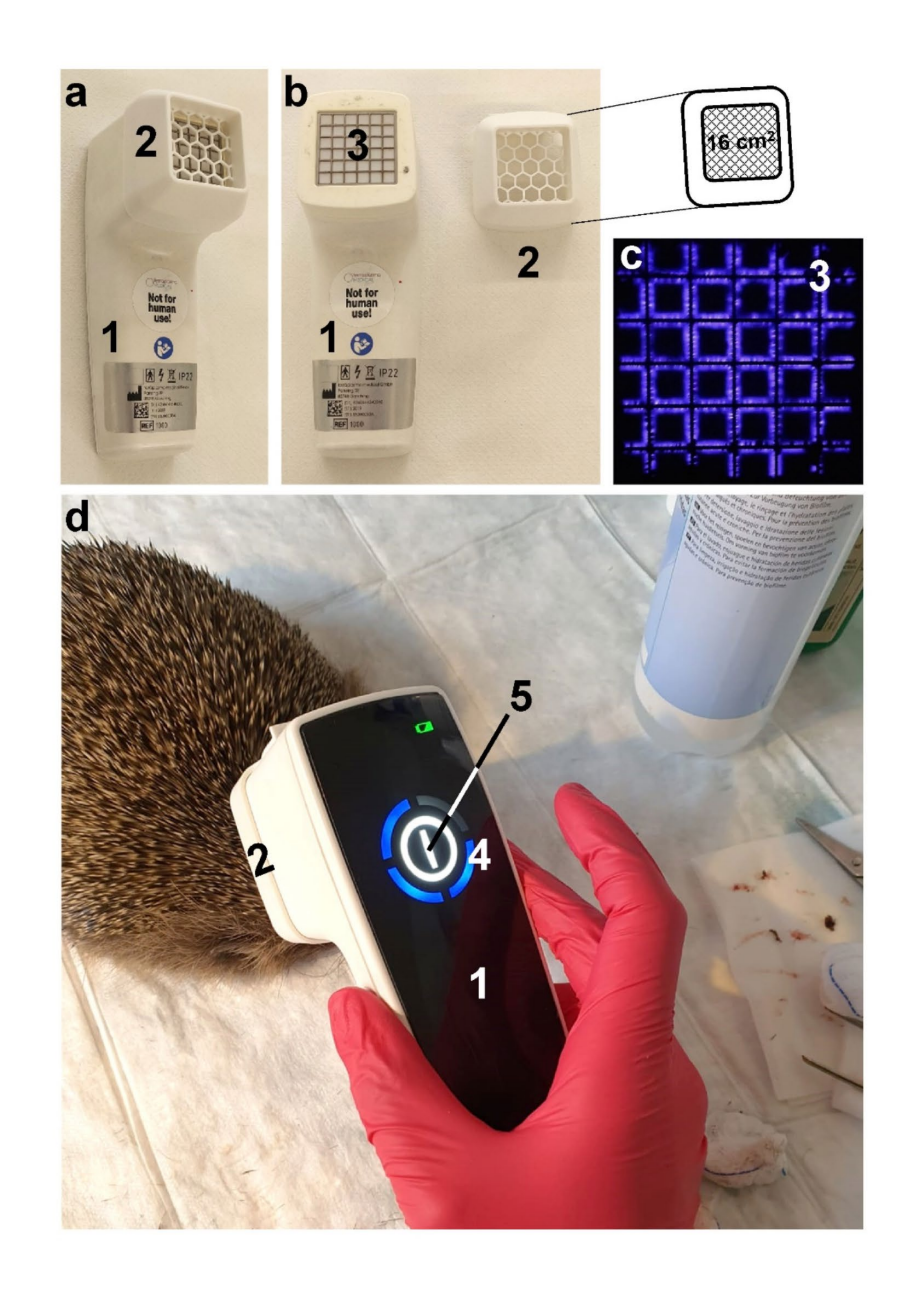
The NIPP device Plasma Care (Terraplasma Medical). (**a**) Ready-to-use Plasma Care device (1) with spacer (2). The opening of the spacer has an side length of 4 cm and thus enables NIPP treatment of a tissue area of 16 cm^2^. (**b**) If the spacer (2) is removed from the Plasma Care device (1), the grid electrode becomes visible (3). (**c**) During operation, NIPP is generated at the grid electrode (3) from the atmospheric ambient air and glows bluish. (**d**) Plasma Care (1) with spacer (2) during patient treatment. During treatment, the internal stopwatch (4) around the start switch (5) runs down and the device switches off automatically after 1 min. For a 2 minutes treatment, the device is started a second time. NIPP is formed from the ambient air in the spacer. The 16 cm2 opening of the spacer is then the interface to the tissue to be treated, where biomedical NIPP effects are induced.

A NIPP device for medical applications (Plasma Care, Terraplasma Medical, Garching near Munich, Germany) was utilized for this purpose (Fig. 2). The Plasma Care device is a battery-operated hand-held device that converts the ambient air into NIPP at the surface of a grid electrode (Fig. 2c). NIPP is formed during operation and fills the volume of the spacer (4.0 cm x 4.0 cm x 1.5 cm; Fig. 2a and **b**). The opening of the spacer (16.0 cm^2^) represents the interface between NIPP and the treated tissue area. For the treatment, the NIPP device was placed with the opening of the spacer on the injured tissue area without pressure. The NIPP generated during operation then diffused through the spacer opening to the surface of the wound.

The biomedical efficacy of NIPP has already been extensively characterized in human medicine. As a result, NIPP has long been used very successfully as an innovative treatment procedure for acute and chronic wounds^29^. Physical plasma is a non-invasive, highly tissue conserving local treatment option. In non-invasive physical plasma (NIPP), gas particles are excited and ionized by applying energy.

In the series of aggregate states solid, liquid and gaseous, mobility, temperature and, in general, energy content of matter continue to increase. NIPP therefore corresponds to a gas to which even more energy has been added until it is ionized. This is why NIPP is also referred to as the 4th state of matter. The excited and ionized NIPP particles, which have a high energy content, also interact with the molecules in the ambient air^30^, resulting primarily in the formation of reactive oxygen species (ROS). These include superoxide anion (O_2_^−^), hydrogen peroxide (H_2_O_2_), hydroxyl radical (HO•), and nitrogen oxides such as nitrogen monoxide (NO), nitrogen dixide (NO_2_), and nitrogen trioxide (NO_3_)^31,32^.

ROS are highly reactive and are the actual biomedically effective factors in NIPP therapy. In contact with tissues and cells, they react with the biomolecules and cell structures and can thus influence physiological processes in tissues. However, since ROS is also produced naturally during cellular processes, for example during metabolic processes, eukaryotic cells have a natural system of antioxidant mechanisms that can detoxify endogenous ROS. This means that NIPP-generated ROS can also be detoxified by the cells. Only when a critical concentration of all ROS is exceeded do NIPP-induced therapeutic effects occur. These depend on the type and phathological condition of the tissue. For example, mammalian skin reacts less sensitively to NIPP than epithelia of internal organs, as skin represents a natural protective barrier against chemical and physical noxae. Similarly, malignant cells are probably more sensitive to NIPP than non-malignant cells, as cancer cells are significantly more metabolically active and therefore show a significantly higher endogenous ROS synthesis. Consequently, ROS detoxification in cancer cells is presumably overloaded more quickly and exogenously supplied ROS can no longer be inactivated. These mechanisms can be therapeutically controlled by adjusting the duration of NIPP treatment. The longer the NIPP exposure lasts, the higher the endogenously supplied, NIPP-induced ROS levels in the tissue. The treatment time thus corresponds to the NIPP dosage^29,30^.

The general redox status as well as individual ROS have signaling functions for the cells and thereby for tissue functionality. Cellular ROS control processes such as cell growth and immune response^28^. Nitrogen-containing ROS such as NO, NO_2_ and NO_3_, for example, are important secondary messengers of digestion, blood flow rate and hypoxia in mammals^18,19^. It is presumably these endogenous properties that make ROS so well tolerated by tissue.

### Additional veterinary care

Regardless of CWM and CWM + NIPP procedures, all patients also required therapies to treat infections and parasite infestations. Depending on diagnosis, antibiotics against bacterial infections (amoxicillin-clavulanic acid, cefovecin, enrofloxacin, metronidazole), antiprotozoals against protozoa (metronidazole, toltrazuril), anthelmintics against endoparasitic nematodes and tapeworms (doramectin, flubendazole, levamisole hydrochloride), acaricides against mites and ticks (allethrin, doramectin, fluralaner), and insecticides against flies, lice, and fleas (allethrin, doramectin, fluralaner) were applied. These therapies were administered independent of the wound healing study and may have lasted longer than the wound healing therapy.

#### Literature analysis

A systematic literature database analysis was carried out to compare the results with the existing literature^33^. Following public online databases were used in accordance with the Preferred Reporting Items for Systematic Reviews and Meta-Analyses (PRISMA; http://www.prisma-statement.org):PubMed(https://pubmed.ncbi.nlm.nih.gov), Scopus (https://www.scopus.com), and Web of Science (https://www.webofscience.com). Following keywords were searched: ‘cold plasma’/‘cold atmospheric plasma’/‘physical plasma’ each in combination with ‘veterinary’/‘animal’. Article title and abstract (Pubmed, Web of Science) or article title, abstract and keywords (Scopus) were searched. The PRISMA display was adapted for the graphical representation of the literature screening.

#### Geography and weather analysis

 The geographic distribution of patient origin was shown using a map of Germany (Federal Agency for Cartography and Geodesy, data license Germany, version 2.0). The map was modified by the authors.

Key weather parameters were included in the analysis and evaluation. These were the air temperature, the amount of precipitation and the duration of sunshine in North Rhine-Westphalia from the years 2021 and 2022. The data was taken from the public online database of the German Weather Service (Deutscher Wetterdienst, https://www.dwd.de).

#### Statistical analysis

 Data presentation and statistical analysis was performed using Prism software (GraphPad Software, San Diego, CA, USA). The mean value, standard deviation and range were specified as statistical parameters. Comparison of the CWM group with the CWM + NIPP group was performed using unpaired two-tailed Welch’s t test. Statistical significance was defined as *p* < 0.05.

## Results

### Patients’ characteristics

 From April 2021 to Aug 2021, 21 patients were included in the CWM group, whereas from May 2022 to Nov 2022, 22 patients were included in the CWM + NIPP group. With a mean estimated age of 2.2 and 2.4 years, the age composition was well comparable in both groups (Table 1). Age information was missing for two patients in the CWM group. Female patients were slightly underrepresented with 6/21 (28.6%; CWM) and 7/22 (31.8%; CWM + NIPP).

**Table 1:** Patients’ characteristics of CWM group and CWM + NIPP group.

Characteristics	CWM	CWM + NIPP
patient number estimated age in years [range]	21 2.2 [1–5]^1^	22 2.4 [1–6]
female [%]	6 [28.6]	7 [31.8]
male [%]	15 [71.4]	15 [68.2]

^1^Two patients without information.

### Injuries to patients and NIPP treatment

 From the injury pattern, bite wounds were more prevalent (CWM: 7/21 = 33.3%, CWM + NIPP: 11/22 = 50.0%) than cut wounds (CWM: 4/21 = 19.1%, CWM + NIPP: 5/22 = 22.7%; Table 2). However, because no information on wound origin was available for 10/21 (47.6%) of CWM patients and 6/22 (27.3%) of CWM + NIPP patients, this statement is not reliable. Wound localization mainly involved the head (CWM: 14/21 = 66.7%, CWM + NIPP: 11/22 = 50.0%). Body (CWM: 3/21 = 14.3%, CWM + NIPP: 7/22 = 31.8%) and extremities (CWM: 4/21 = 19.0%, CWM + NIPP: 4/22 = 18.2%) were significantly less affected. In the CWM + NIPP group, NIPP treatment was given every 3 days from day 1 until wound care was completed. In the majority of CWM + NIPP patients, NIPP treatment three or four times was sufficient until successful completion of wound care (9 + 7/22 = 72.7%). The remaining patients were treated with NIPP five times (2/22 = 9.1%), six times (1/22 = 4.6%), and seven times (2/22 = 9.1%), respectively. For one patient in the CWM + NIPP group, there exists no documentation on the number of NIPP treatments (1/22 = 4.6%).

**Table 2:** Injury patterns of patients in the CWM group and the CWM + NIPP group including the number of NIPP treatments in the CWM + NIPP group.

Characteristics	CWM	CWM + NIPP
bite wound [%]	7/21 [33.3]	11/22 [50.0]
cut wound [%]	4/21 [19.1]	5/22 [22.7]
wound origin unknown [%]	10/21 [47.6]	6/22 [27.3]
head wounded [%]	14/21 [66.7]	11/22 [50.0]
body wounded [%]	3/21 [14.3]	7/22 [31.8]
extremities wounded [%]	4/21 [19.0]	4/22 [18.2]
NIPP treatment three times^1^	-	9/22 [40.9]
NIPP treatment four times	-	7/22 [31.8]
NIPP treatment five times	-	2/22 [9.1]
NIPP treatment six times	-	1/22 [4.6]
NIPP treatment seven times	-	2/22 [9.1]

^1^NIPP treatment was not documented for 1/22 (4.6%) patient in the CWM + NIPP group.

#### Spatial distribution of the recruited patients

**Fig. 3:**
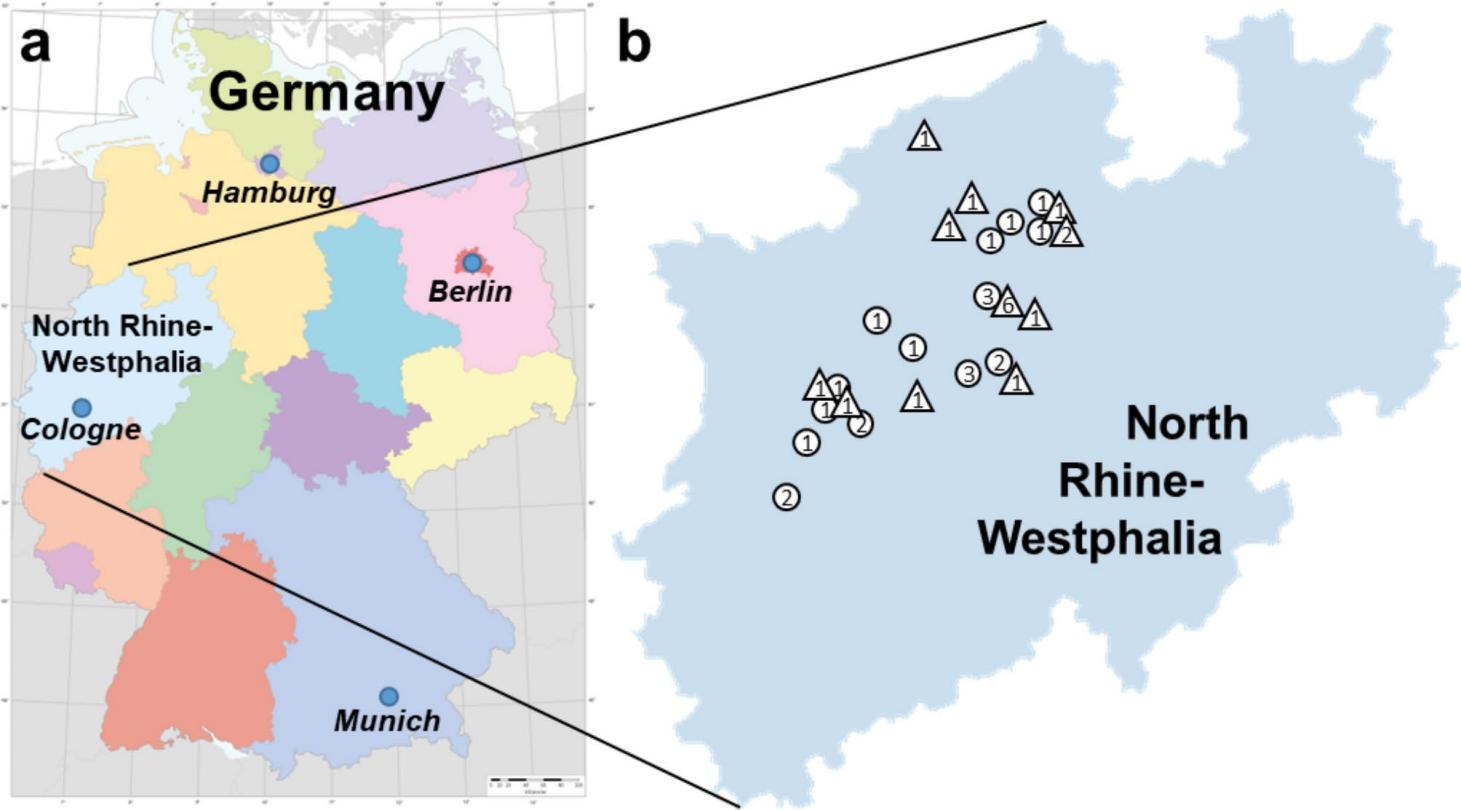
Patient origins. (**a**) All patients are from the German state of North Rhine-Westphalia. (**b**) Distribution of CWM group (*n* = 21; circles) and CWM + NIPP group (*n* = 22; triangles) patients. Numbers in the symbols indicate the number of patients. For 5/22 (22.7%) patients of the CWM + NIPP group no site of origin is documented.

 All patients in the clinical trial are from western Germany and are all located within the federal state of North Rhine-Westphalia (Fig. 3). These were urban and suburban cultural landscapes with pronounced human settlement. The sites were distributed relatively evenly across the the north-western half of North Rhine-Westphalia. The spatial distribution of the 2021 sites was approximately the same as the distribution from 2022. Thus, regional differences between patients in the CWM group and the CWM + NIPP group were not expected. The origin of 5/22 (22.7%) patients of the CWM + NIPP group was not documented.

#### Seasonal distribution of the recruited patients

**Fig. 4:**
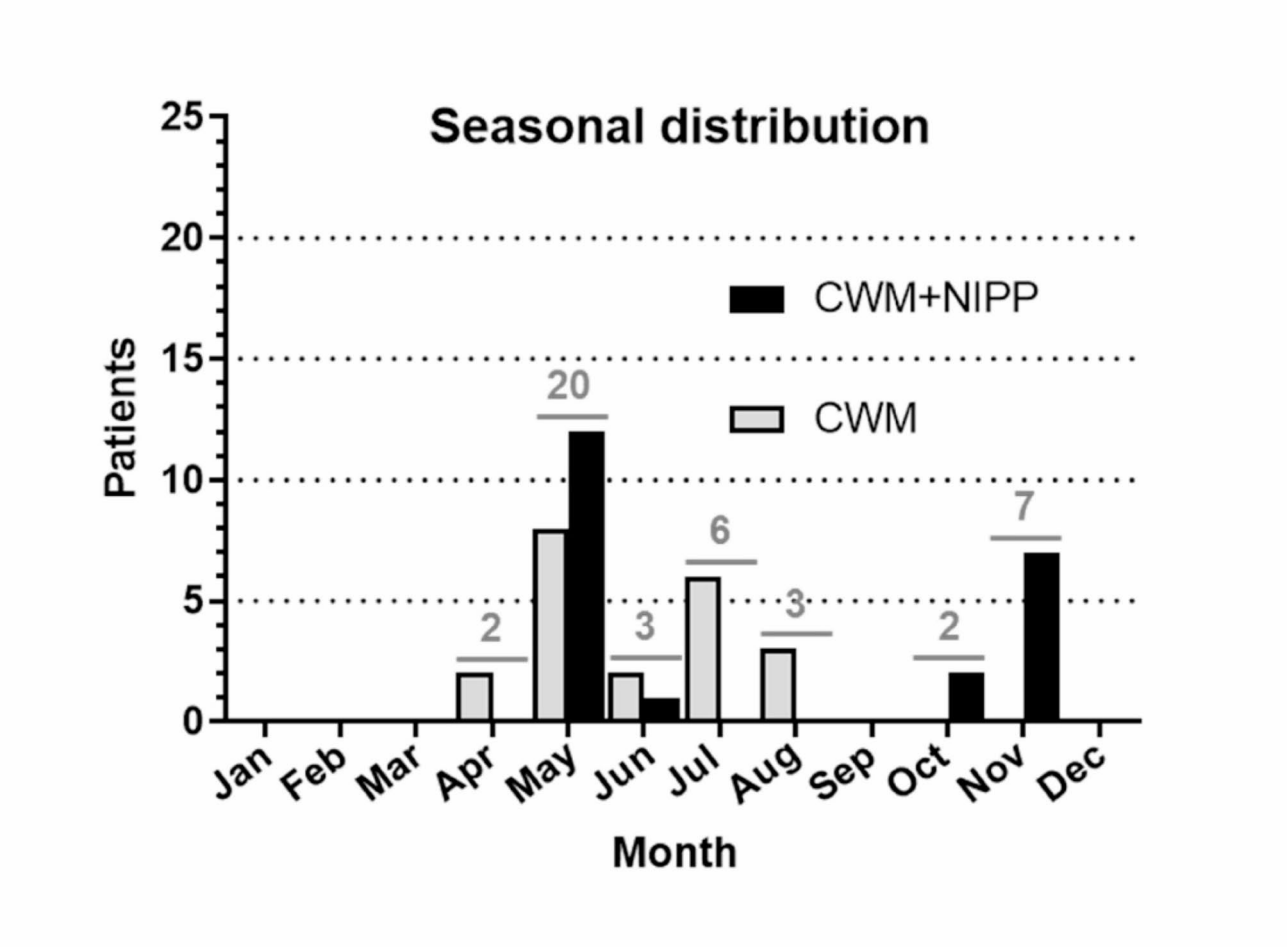
Seasonal distribution of CWM patients (light gray) and CWM + NIPP patients (black). Shown are all patients found and delivered to the Wildlife Rescue Center from April 2021 to Nov 2022. The total number of patients delivered in which month is shown in dark gray above the CWM and CWM + NIPP columns.

 While the spatial distribution of both groups was comparable, there were differences in the seasonal distribution. Recruitment of the 2021 CWM patients occurred rather evenly over the months of April through August (Fig. 4). In contrast, the patients in the CWM + NIPP group were enrolled mainly in May (12/22 = 54.6% patients) and November 2022 (7/22 = 31.8% patients). 1/22 (4.6%) and 2/22 (9.1%) patients were added in June and October 2022, respectively. These two recruitment foci of the CWM + NIPP group in the middle and end of the year do not change when the four euthanized CWM + NIPP patients are taken into consideration. These were recruited twice in May, once in October, and once in November. For the CWM group, the 4 euthanized patients rather confirm the nearly even seasonal distribution from April to August (April: 1 patient, May: 2 patients, July: 1 patient).

The habits and general activity of animals depend largely on the weather conditions. The summer of 2022 was warm and dry in North Rhine-Westphalia and in Germany as a whole. Compared to 2021, the annual average air temperature in North Rhine-Westphalia in 2022 was 1.4 K higher and there were significantly more hours of sunshine (1984.0 h) than in the previous year (Table 3). This was accompanied by a clearly lower amount of precipitation (2021: 840.7 l/m2; 2022: 715.8 l/m2).

**Table 3:** Annual average of ambient air temperature, precipitation rate and hours of sunshine in North Rhine-Westphalia in 2021 and 2022 (data from: Deutscher Wetterdienst; https://www.dwd.de).

Climatic parameter	2021	2022
ambient air temperature [°C] precipitation rate [l/m^2^]	9.8 840.7	11.2 715.8
hours of sunshine [hours]	1508.4	1984.0

#### Improvement of wound healing by combining NIPP treatment with CWM compared to CWM only

**Fig. 5:**
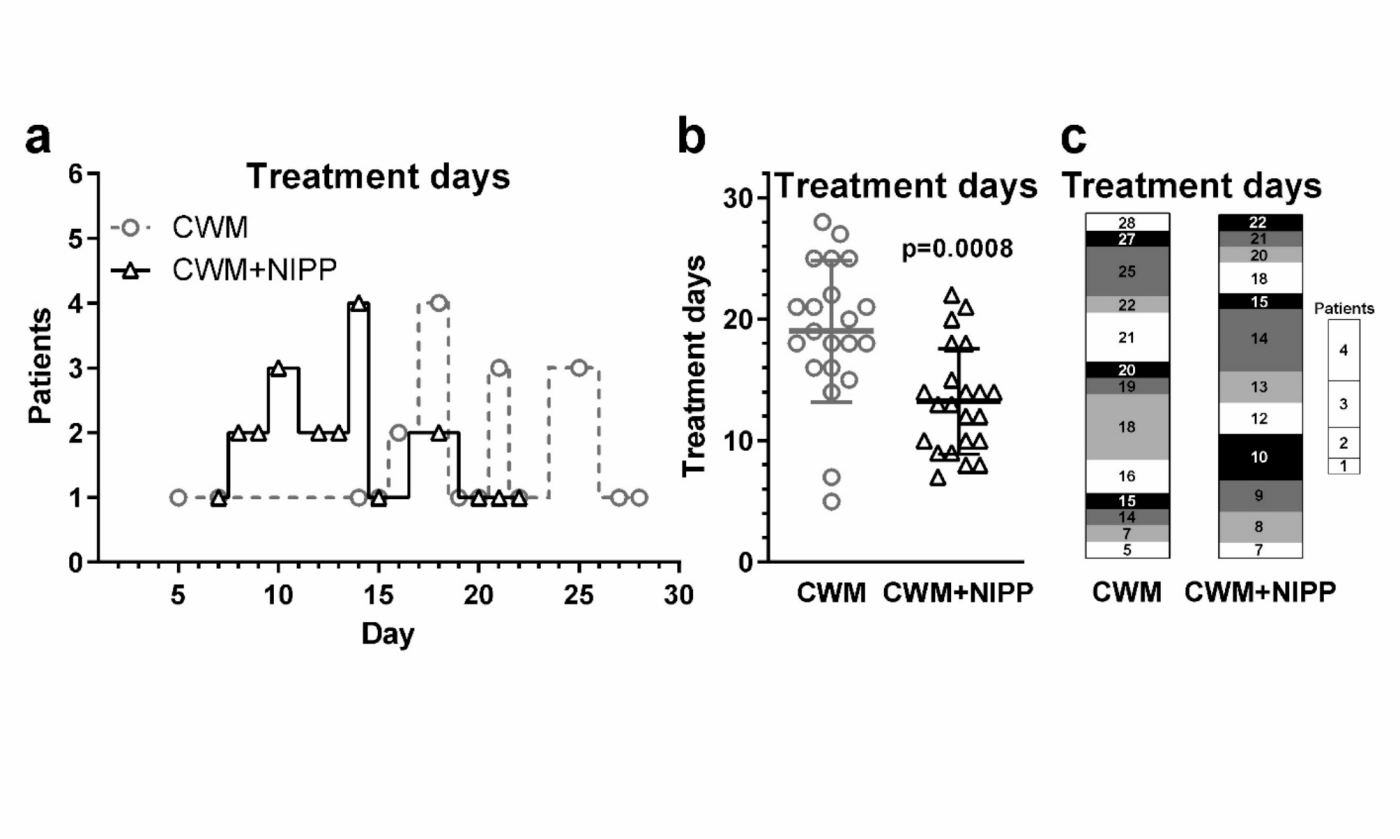
Comparison of CWM versus CWM + NIPP wound treatment. (**a**) Distribution of wound treatment duration in days. Plotted is the number of patients per required wound treatment in days for the CWM group (gray dashed line, circles) and the CWM + NIPP group (black line, triangles). (**b**) Plot illustration and statistical comparison of mean values of wound treatment days of CWM group and CWM + NIPP group. Significance was calculated using Welch’s t test. (**c**) Stack plot of the wound treatment duration in days. The height of the segments (marked off from each other in white, light gray, dark gray and black) indicates the number of patients as shown in the legend.

 The patients in the CWM-NIPP group were additionally treated with NIPP for 2 min every 3 days. As a result, wound treatment was completed significantly earlier in this group than in the CWM group without NIPP treatment (Fig. 5). Wound care in the CWM group required 5 to 28 days, with a statistical average of 19.0 days. In contrast, patients in the CWM-NIPP group were treated for an average of only 13.2 days (range 7–22 days). This difference of almost 6 days was statistically significant (*p* = 0.0008).

Origin, location and area of the wounds were not taken into account in the analysis as they were not documented.

The comparability of the two cohorts CWM and CWM + NIPP including NIPP treatment results are summarized in Table 4.

**Table 4:** Summarized comparison of the patient groups CWM vs. CWM + NIPP and outcomes of the clinical wildlife trial.

Parameters	CWM vs. CWM + NIPP comparison
Patiens’ characteristics	comparable
Injury patterns	comparable
Geographical distribution	comparable
Seasonal distribution	different
Climatic factors	slightly different
Wound treatment duration	19.0 d (CWM) vs. 13.2 d (CWM + NIPP)^1^

^1^Statistically significant (*p* = 0.0008).

##### Systematic literature database search

**Fig. 6:**
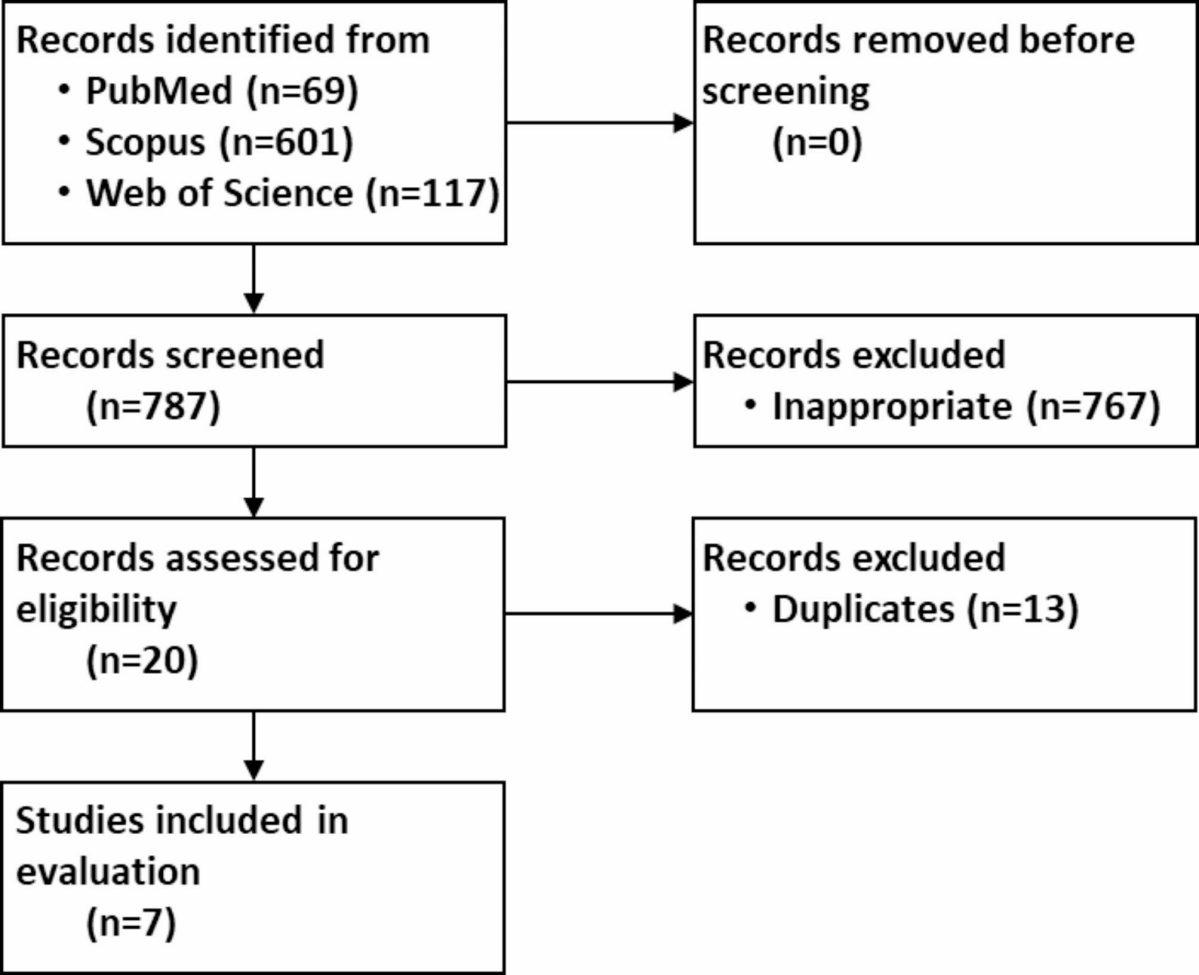
Systematic literature database search on NIPP therapy in veterinary medicine. For details on the search parameters, please refer to the ‘Patients and Methods’ section.

 Three public literature databases were searched in order to place the present study in the context of the existing literature on veterinary NIPP applications. The search terms ‘cold plasma’, ‘cold atmospheric plasma’, and ‘physical plasma’ were used in combination with the keywords ‘veterinary’ and ‘animal’. The search delivered 69 (PubMed), 601 (Scopus), and 117 (Web of Science) hits (Fig. 6).

Out of a total of 787 hits, 767 hits were classified as inappropriate. The off-topic articles mostly dealt with NIPP applications on laboratory animals or in the area of food production and processing. The 20 articles contained 13 duplicates. Thus, a total of 7 articles on NIPP applications in veterinary medicine were found using the search parameters applied (Table 5).

**Table 5:** Results of the systematic literature database search on NIPP in veterinary medicine.

Year	Article type	Species	Disease	Results	Reference
2017	Case report	Canine (*n* = 1)	Cutaneous *Alternaria spp.* infection	Complete clinical remission	^34^
2018	Case report	Snake (*n* = 1)	Severe bacterial pyoderma	Reepithelialization and complete healing	^35^
2021	Review	-	Focus on wound healing and experimental animal models	-	^36^
2022	Case report	European Hedgehog (*n* = 1)	Acute wound	Accelerated healing	^28^
2023	Review	-	Focus on infectious diseases	-	^37^
2023	Case report	Feline (*n* = 1)	Head and neck squamous cell carcinoma	Response in 2 of the 3 treated tumors	^38^
2023	Clinical trial	Feline (*n* = 4), Canine (*n* = 23)	Acute and chronic wounds	Complete healing in 81.5% of cases	^39^

## Discussion

The skin of vertebrates protects the organism from microbial, physical and chemical noxae. Skin injuries can be healed by regeneration or repair. During skin repair, missing tissue sections are filled with fibrotic tissue, which leads to scar formation and functional restrictions of the tissue. In skin regeneration, on the other hand, skin cells migrate into the lesion and reconstruct the original skin architecture. Therefore, the original physiological functionality is almost completely preserved^40^. In higher vertebrates, injured skin is healed almost exclusively by repair. Regenerative processes only occur in embryonic skin. In amphibians, skin injuries are also closed regeneratively in adult individuals^41^. Ageing processes of the skin are determined by intrinsic processes (e.g. mesenchymal stem cells) and external environmental factors (e.g. UV radiation)^42^.

Wound healing is widespread in multicellular organisms, but mechanisms for tissue renewal differ considerably even in closely related animal groups. Wound healing closes the wound and thus prevents infection. Cutaneous wound healing in mammals is controlled by growth factors, cytokines, matrix components and immune cells (monocytes, lymphocytes, macrophages, mast cells). Tissue-specific cells (fibroblasts, epithelial cells, endothelial cells) are also involved locally. During healing, a complex remodeling of the extracellular matrix takes place, which supports the formation of new structural elements^43,44^.

Wound healing in vertebrates is generally categorized into three phases. These can overlap or occur simultaneously. During the inflammatory phase, a local immune response is initiated to protect against infection. This is followed by the proliferation phase with repair and regeneration processes, angiogenesis and reepithelialization. Finally, in the maturation or remodeling phase, the extracellular structures are adapted. This also leads to scar formation^44,45^.

Veterinary wound management is complex and individual, but there is a generally accepted standard concept. The main principles are debridement, wound irrigation and antibiotics^46^. Other treatment options exist to support the healing process. Newly developed wound dressing materials absorb excess exudate and protect against dehydration and maceration. Biological and biocompatible polymers such as collagen, hyaluronic acid, alginates and hydrogels with biologically active substances are also applied^47^.

The combination of CWM and NIPP application can be integrated into this multifactorial concept. ROS generated during NIPP treatment alter redox status and pH of the tissue, for example through the formation of nitric acid from dissolved NO_3_^−^. Furthermore, some ROS also act as secondary messengers and regulate antibiosis, immune response and cellular regeneration^17,19,48^. In human medicine, NIPP has been used intensively for wound healing since the 2000s, and the data in the literature is clearer^25,49–51^.

The NIPP device Plasma Care (Terraplasma Medical) is established in human medicine for the treatment of dermal infections, wounds, and infected wounds. A diabetic mellitus patient with severe *Trichophyton rubrum*infection of the toenails and areas of the foot (onychomycosis) did not respond to topical antifungal ointment (efinaconazole plus luliconazole). After 1 week of daily NIPP treatment, the microbiological analysis was negative^52^. In patients with chronic hard-to-heal wounds, a reduction in infections was also demonstrated^53^. Furthermore, NIPP treatment with the Plasma Care device reduced the wound area by 76%. One NIPP effect was the activation of granulocytes and increased granulation^53,54^. Finally, a phase I study with 28 patients and the following phase II study with 64 patients showed a reduction of radiodermatitis in fractionally irradiated breast cancer patients^55–57^.

Due to its compact design, the NIPP device was very easy to use on the injured patients. Injured areas of the European hedgehog’s skin could be reached easily despite the quills. The device is very quiet in its operation, does not vibrate, does not heat up and produces no gas or NIPP flow. The patients showed no signs of restlessness or even pain during the treatment and did not try to escape. Anesthesia was not administered. This may rather suggest that the NIPP treatment did not cause much discomfort or pain to the patients. This assumption is supported by NIPP therapies in human patients. Depending on the area of the body treated, patients describe the NIPP application as pain-free or at most report a slight stinging sensation^58–61^. As a result, the NIPP Plasma Care device and NIPP therapy in general appear to be very well suited for the wound treatment of animals. Due to the short treatment period in the care of the wildlife rescue center, no conclusive statements can be made about potential side effects of NIPP treatment. However, no acute adverse treatment effects were observed during the observation period of the clinical trial.

There is little published data on the use of NIPP in veterinary medicine. A systematic literature search in the public databases PubMed, Scopus, and Web of Science identified seven suitable hits. Independent of this systematic search, one article in the journal ‘Clinical Plasma Medicine’, published from 2013 to 2020^62^, one book chapter on NIPP therapy in veterinary medicine^63^and two articles in unlisted veterinary journals^64,65^were also found. The systematic literature search revealed only one veterinary clinical study. Like the present trial, this also dealt with wound management. Yoo et al. treated the wounds of 27 cats and dogs with NIPP in 2023. However, the setup included patients treated after CWM and both acute and chronic wounds were included^39^. The overall healing rate was 81.5% and NIPP therapy was administered for up to 7 weeks. Although the design of this clinical trial differs from the present examination, the basic findings are comparable. All other published veterinary NIPP applications are limited to experimental studies on laboratory and large animals or were carried out as individual single treatments^28,34–37,62–65^.

The presented study was designed as a prospective, non-randomized monocentric clinical trial. The geographical catchment area of the injured European hedgehogs covered approximately the north-western third of North Rhine-Westphalia. The geographical distribution of the patients in the two groups was distributed evenly across this area. Analysis of patient characteristics also revealed a relatively homogeneous structure of the two study groups. The number of patients and estimated age were very similar in the CWM and CWM + NIPP group. The ratio of bite to cut wounds were also similar. However, in the CWM group the origin of the wound could be determined somewhat less clearly. Both groups were also very similar in terms of injury localization. The head was mainly affected by bites or cuts. There were about three times more males than females in both groups. This is possibly due to the fact that male European hedgehogs have a larger action radius than females, especially during the mating season. As a result, males are presumably more exposed and have an increased risk of injury^66,67^. In addition, European hedgehogs are increasingly fed by humans in urban areas. If males meet at artificial feeding sites, this can lead to conflicts, including injuries.

The trial period began in April and May. This is the period in which European hedgehogs awaken from hibernation and enter the reproductive phase^68^. The activity is correspondingly high, especially among males. During the rest of the year until November, the rate is more or less constant. However, it remains striking that no patients appear in the CWM + NIPP group from July to September. This fact cannot be explained by the NIPP treatment itself. The most likely explanation is environmental factors, e.g. weather conditions, which may have differed between the two seasons of the study period. The summer of 2022 was warmer and drier in North Rhine-Westphalia than in 2021. These factors could have led to the European hedgehogs being less active in the summer of 2022 than in 2021^67^. On the other hand, the drier summer of 2022 is also likely to have meant that lawns and hedges were trimmed less frequently. This in turn could mean that the risk of European hedgehogs being injured by garden tools would be reduced. Furthermore, the patients in the CWM + NIPP group were exposed to the cooler climatic conditions of early summer and fall at the time of injury. It can therefore not be excluded that weather factors also had an influence on wound healing and thus on the trial findings.

It is also possible that the seasonal differences in 2021 and 2022 are due to the small cohort size of the two groups. Larger European hedgehog wildlife studies demonstrated an almost Gaussian distribution of patient numbers with a peak in the summer months^69^. The small number of patients included depends on how many injured wild animals are handed in to the wildlife rescue center. Compared to clinical trials in veterinary institutions or in human medicine, this number is significantly lower and can hardly be influenced. Furthermore, the admission and supply capacity of the wildlife rescue center must also be taken into account. It can occur that all medical treatment places are occupied and further injured patients have to be rejected. Several institutions could collect data in a multicenter follow-up trial. The small number of patients weakens the statistical significance of the present monocentric clinical trial.

The treatment of comorbidities, e.g. infections and parasitic infestations, may also correlate with wound healing. The clinical trial presented demonstrates that all injured patients required additional antimicrobial and antiparasitic treatment. In 2006, adverse drug interactions in veterinary medicine were analyzed in Switzerland. It was found that 190 suspected adverse events were reported due to the use of veterinary drugs^70^. These were mainly drugs against parasites, e.g. helminths, protozoa, and parasitic fungi (‘anti-parasitics’; 48%) and microorgansims like bacteria, viruses, and infectious fungi (‘anti-infectives’; 20%). This was confirmed in a Swedish study from the observation period 1991–1995^71^. However, this examination demonstrated that the adverse drug reactions were mainly attributed to anti-infectives, while anti-parasitics hardly influenced them at all. There is a little data on the interaction of NIPP treatment and drugs or disinfectants. Studies on injured sheep and dogs showed that NIPP alone had no advantages over treatment with polyhexanide. In contrast, the combination of NIPP + polyhexanide demonstrated a higher efficacy^62,65^. It could therefore be assumed that NIPP wound healing efficacy is best achieved in combination with CWM. Another problem with the antibiotic components of CWM is possible antibiotic resistance, drug interaction and adverse drug effects^72,73^. Here, too, it could be assumed that the administration of NIPP can reduce antibiotics with all the associated negative effects.

Finally, the duration of wound treatment also depends to a large extent on wound severity, wound area, wound depth and the number of injured areas. For practical reasons, these parameters could not be collected in this clinical trial. The medical history, e.g. age, origin of the wound, was also only estimated or assumed. A conceivable increase in stress or pain during the NIPP treatment could not be assessed using laboratory parameters. Conclusions on this were derived from the behavior of the animals and are therefore only of limited significance. These limitations, in particular the small cohort sizes, certainly contribute to a certain statistical bias in the results obtained.

Despite all the limitations of a clinical wildlife trial, there is evidence of the veterinary efficacy of NIPP application in wounds. The wound treatment of patients in the CWM + NIPP cohort was completed on average 6 days earlier than that of patients in the CWM cohort without additional NIPP application (CWM + NIPP: 13.2 days vs. CWM: 19.0 days). This treatment benefit is most likely at least partly due to the NIPP treatment and was statistically significant (*p*= 0.0008). Thus, the clinical trial confirms the initial results of a single case treatment^28^ and could potentially be considered a promising option for wound management in animals in general. In addition to European hedgehogs, this would apply to all wild mammals as well as zoo, domestic and farm animals. We can only speculate about the treatment of injured non-mammals due to a lack of data. However, the biomedical principles of action make wound-healing effects in other vertebrates appear quite likely.

In 72.7% (16 of 22) of the CWM + NIPP cases, three (9 patients) or four (7 patients) NIPP treatments were sufficient to complete the wound treatment. Only 5 patients underwent NIPP treatment five, six or seven times. The frequency of treatment presumably plays no role with regard to possible undesirable side effects. In vitro and in vivo studies have shown that the application of NIPP to human and mouse skin has virtually no effect on cell physiology, cytology or genome stability^57,74–78^. NIPP application should therefore also be safe in other mammals. However, it would be desirable to keep the overall duration of wound therapy as short as possible. This would reduce costs and make it possible to release the patients back into the wilderness quickly.

Despite the results of the clinical trial, some questions remain unanswered. In the study presented here, a 2-minute NIPP treatment was carried out uniformly every three days. Both the treatment duration and frequency should be evaluated and adjusted if necessaray to achieve an optimal wound healing effect. Furthermore, wound type, deepth, and area should be included in the analyses in larger cohorts. It would also be important to include further clinical parameters such as wound moisture and infection status.

## Conclusions

The NIPP device Plasma Care (Terraplasma Medical) was demonstrated to be suitable for wound treatment of European hedgehogs. The patients exhibited no behavior of stress, discomfort or pain. Not even after repeated treatments with the NIPP device. Compared to the CWM control group, wound treatment was completed statistically significantly 6 days earlier in the NIPP-treated patients of the CWM + NIPP group. This reduces the stress for the wildlife patients and can reduce the amount of material required and thus the costs for the wildlife rescue center. This clinical wildlife study indicates the possibility of using NIPP for wound healing in wild European hedgehogs. Potentially, other wildlife as well as domestic and farm animals would also benefit from NIPP applications in the treatment of injuries.

## Data Availability

All data are given in the article and can be obtained from the corresponding author in electronic form upon justified request.
